# Assessing the cost-effectiveness of annual COVID-19 booster vaccination in South Korea using a transmission dynamic model

**DOI:** 10.3389/fpubh.2023.1280412

**Published:** 2023-11-23

**Authors:** Wongyeong Choi, Eunha Shim

**Affiliations:** Department of Mathematics, Soongsil University, Seoul, Republic of Korea

**Keywords:** COVID-19, coronavirus disease, COVID-19 vaccine, annual vaccination, South Korea, cost-effectiveness, mathematical modeling, SARS-CoV-2

## Abstract

**Introduction:**

We evaluated the cost-effectiveness of South Korea’s planned annual coronavirus disease 2019 (COVID-19) booster campaign scheduled for October 2023.

**Materials and methods:**

An age-structured mathematical model was used to analyze the public impacts and cost-effectiveness of vaccination across three vaccination strategies: uniform allocation and prioritizing those over 65 or those over 50 years old. We calculated the incremental cost per quality-adjusted life year (QALY) from both healthcare and societal perspectives. The maximum vaccine cost for cost-effectiveness was also identified.

**Results:**

Our analysis highlights the cost-effectiveness of South Korea’s annual COVID-19 vaccination program in mitigating health and economic impacts. The most cost-effective strategy is uniform vaccine allocation, offering the lowest incremental cost-effectiveness ratio (ICER) at US$ 25,787/QALY. However, with a relatively high attack rate, the strategy prioritizing individuals over 65 years emerges as more cost-effective, lowering the ICER to US$ 13,785/QALY. Prioritizing those over 50 was less cost-effective. All strategies were cost-saving from a societal perspective, with cost-effectiveness being more sensitive to vaccine price than to its effectiveness.

**Discussion:**

Our results imply a potential strategy shift in current vaccination plan, with uniform vaccine distribution being more cost-effective than prioritizing older adults. Early estimation of viral transmissibility and vaccine effectiveness is crucial in determining the most cost-effective vaccine allocation approach.

## 1 Introduction

The ongoing COVID-19 pandemic poses substantial public health and economic challenges globally. In South Korea alone, over 33.8 million COVID-19 cases and 35,522 related fatalities have been reported as of August 16, 2023 (1). In light of this, South Korea has employed several preventive measures such as self-isolation, contact tracing, mask usage, social distancing, and notably, vaccination. Since its initiation in February 2021, the vaccination program has significantly mitigated the COVID-19 burden, averting an estimated 143,000 deaths (2). Initial vaccine uptake rate was relatively high in South Korea, and approximately 94.2% of the eligible population had completed their primary vaccine series within the first year (3). However, a decline in the uptake of booster doses has emerged, attributable to vaccine hesitancy and fatigue, resulting in a 15.2% coverage among the eligible population as of April 5, 2023 (3).

On March 22, 2023, the country announced its transition from mass vaccination to an annual booster program for the general population, with a focus on minimizing COVID-19-related deaths and hospitalizations (2). The government plans to offer free COVID-19 vaccines targeting the omicron sub-variant XBB.1.5.2 for all individuals over 6 months of age starting in October 2023 (2). Additionally, this program prioritizes high-risk groups, including individuals aged over 65, employees and residents of long-term care facilities, and immunocompromised individuals (2).

However, the potential implications of this annual COVID-19 vaccination program are currently under debate (4, 5). Unlike seasonal influenza, COVID-19’s lack of consistent seasonal patterns and the continuous emergence of new variants introduce uncertainty regarding vaccine effectiveness (4, 5). In this context, mathematical modeling could shed light on the epidemiological and economic impacts of annual vaccination strategies. Prior studies have indicated the cost-effectiveness of COVID-19 vaccination—even in low- and middle-income countries—and have underscored the potential cost-effectiveness of administering the third and fourth booster shots, as well as vaccines with reduced efficacy against variants such as Omicron (6–8). Moreover, a previous South Korean study has emphasized the importance of rapid vaccination to decrease the overall costs associated with COVID-19 during the 2021–2022 outbreak (9).

Considering the evolving nature of the COVID-19 pandemic, it is crucial to evaluate the epidemiological and economic impacts of various annual vaccination strategies. In this study, we utilize an age-structured mathematical model of SARS-CoV-2 transmission and vaccination to assess the cost-effectiveness of annual vaccination programs in South Korea. This assessment will consider various scenarios, including vaccination prioritization, vaccine price, and the potential emergence of highly transmissible subvariants.

## 2 Materials and methods

### 2.1 Study design

In this cost-effectiveness study, we compared the annual COVID-19 vaccination program in South Korea to a scenario where no further vaccinations are administered. We utilized an age-structured dynamic model of COVID-19 transmission to examine the intricate interactions between various age groups and the spread of the virus. This model was subsequently incorporated into a cost-effectiveness analysis framework to appraise the economic merit of the vaccination programs.

The benefits of vaccination strategies were measured as a reduction in disease burden and the corresponding increase in quality-adjusted life years (QALYs). A baseline analysis, viewed from a healthcare perspective, examined the direct costs and health consequences related to the vaccination program. Additionally, a sensitivity analysis was undertaken from a societal perspective, evaluating a wider range of costs and benefits extending beyond the healthcare sector.

Our cost-effectiveness analyses spanned a period of one year to encapsulate the immediate impacts of the annual vaccination program. By comparing the costs incurred and the QALYs gained through vaccination, this study aims to provide essential insights into both the economic and epidemiological effects of the vaccination program.

### 2.2 Age-structured model of COVID-19 transmission and vaccination

**Figure 1:**
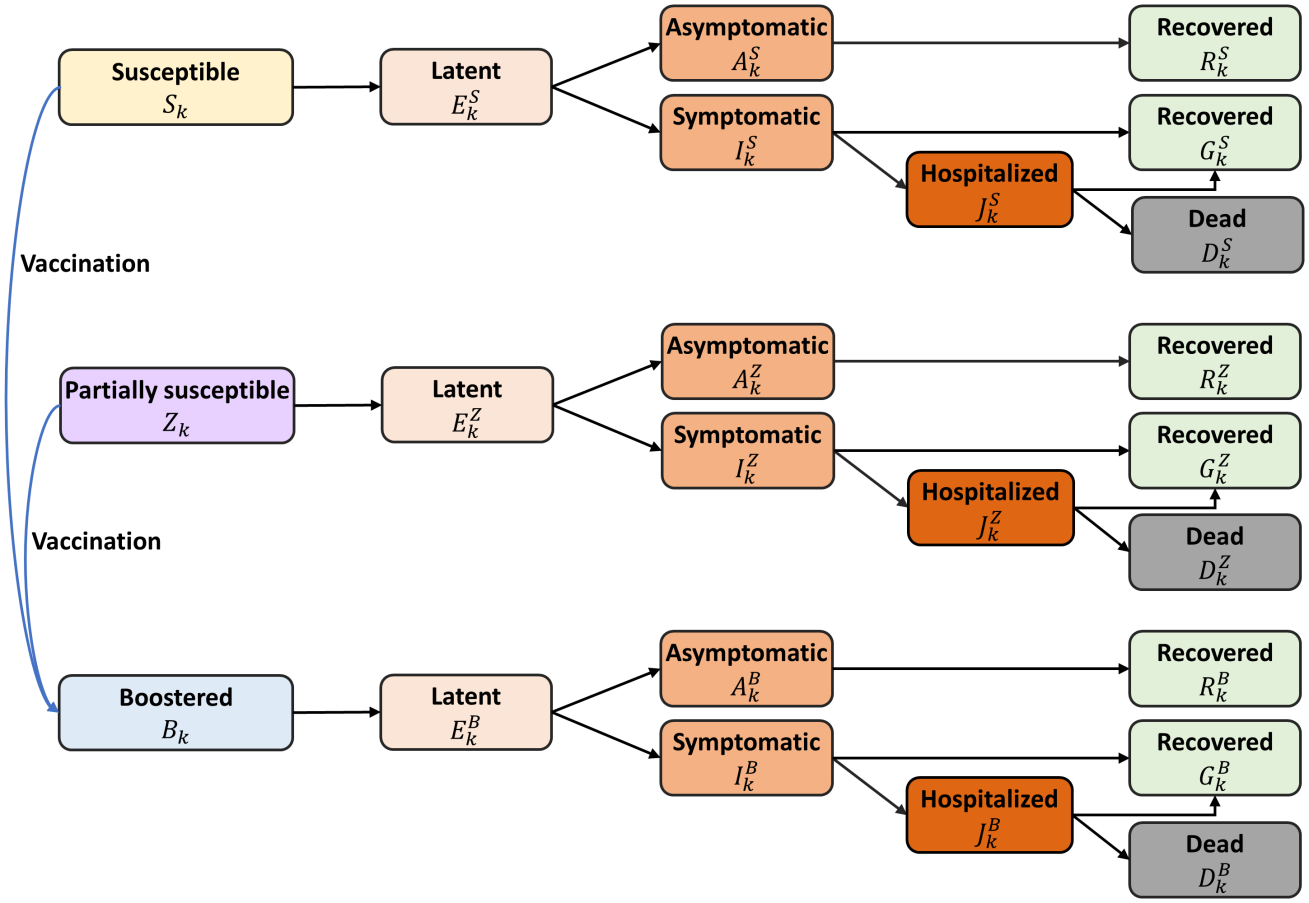
Diagram of the mathematical model of COVID-19 transmission with annual vaccination. All individuals were stratified by age and epidemiological status. The model variables are denoted as follows: fully susceptible ( $S_{k}$ ), partially susceptible due to hybrid immunity ( $Z_{k}$ ), vaccinated under the annual program ( $B_{k}$ ), latent ( $E_{k}^{x}$ ), infectious but asymptomatic ( $A_{k}^{x}$ ), infectious with symptoms ( $I_{k}^{x}$ ), hospitalized ( $J_{k}^{x}$ ), recovered from asymptomatic infection ( $R_{k}^{x}$ ), recovered from symptomatic infection ( $G_{k}^{x}$ ), and deceased from the disease ( $D_{k}^{x}$ ), with superscript ${}_{x}$ representing immune status: fully susceptible ( x=S ), partially susceptible ( x=Z ), and vaccinated under the annual program ( x=B ). The detailed descriptions of model variables and parameters were represented in Supplementary material.

We formulated a deterministic compartmental model incorporating SARS-CoV-2 transmission dynamics, vaccination, and pre-existing immunity against COVID-19 (Figure 1). In South Korea, 70.0% of the population, from 47.6% of those aged over 80–90.0% of those aged between 5 and 9, were found to have N-antibodies, an indicator of natural infection (2). This implies that approximately two-thirds of the South Korean population had been infected with COVID-19, a premise which formed the basis for the initial conditions of our mathematical model.

We divided the population in the model into distinct 17 age groups: under 6 months, 6 months–4 years, 5-year intervals from age 5 to 74, and those over 75 years. The model did not account for natural births or deaths, given the relatively short simulation time, one-year. Within each age group, individuals were further classified by their epidemiological status, including fully susceptible individuals ( $S_{k}$ ), those partially susceptible due to hybrid immunity ( $Z_{k}$ ), those vaccinated under the annual program ( $B_{k}$ ), and categories that depict various stages of infection and recovery: latent ( $E_{k}^{x}$ ), infectious but asymptomatic ( $A_{k}^{x}$ ), infectious with symptoms ( $I_{k}^{x}$ ), hospitalized ( $J_{k}^{x}$ ), recovered from asymptomatic infection ( $R_{k}^{x}$ ), recovered from symptomatic infection ( $G_{k}^{x}$ ), and deceased due to the infection ( $D_{k}^{x}$ ). The superscript x refers to immune status: fully susceptible ( x=S ), partially susceptible ( x=Z ), and vaccinated under the annual program ( x=B ).

The infection rate among susceptible individuals, represented as $\lambda_{k}\left(t\right)$ , was influenced by various factors such as age-specific infection probability upon contact with an infectious person, the frequency of daily contacts with different age groups, and the proportion of infectious individuals in each age group. Following an infection, we presumed a latency period of 3.1 days regardless of the immune status, whereas the probability of manifesting symptomatic disease was contingent on age (10, 11). For simplicity, our model assumed symptomatic cases would be reported and managed as outpatients, and only (partially) susceptible individuals could benefit from vaccination. All infected individuals were presumed to be equally contagious, and a fraction of those with symptoms were projected to require hospitalization. Hospitalized individuals, after an average stay of 11 days, would either recover or die from the disease, at age-specific rates (12). Baseline values and ranges of epidemiological parameters employed in the simulation are given in Supplementary Table S1. Further details of the model are provided in the Supplementary material.

### 2.3 Vaccine effectiveness

We hypothesized that both vaccine-induced and infection-induced immunity would decrease susceptibility to COVID-19 infection and lower the chance of severe or critical illness. We posited that individuals with hybrid immunity ( $Z_{k}$ ), who have antibodies induced by both infection and vaccination, would demonstrate a stronger immune response than those fully susceptible. In our model, this bolstered immune response is calculated as a 14.6% protective effectiveness against infection ( $\sigma_{Z}$ ), and the decrease in the likelihood of severe illness leading to hospitalization is denoted as $\omega_{Z}$ , relative to fully susceptible individuals. This assumption is in line with evidence indicating the substantial and long-lasting protective effects of hybrid immunity against SARS-CoV-2 variants (13). The proposed linear decline in the protective effect provided by hybrid immunity with primary doses is based on the estimated effectiveness against reinfection at 3 months (69.0% with a 95% CI: 58.9–77.5%) and at 12 months (41.8% with a 95% CI: 31.5–52.8%) (14). The protective effectiveness against hospitalization ( $\omega_{Z}$ ) for partially susceptible individuals is presumed to be 0.31, which is derived from the relative risk of hospitalization for those with hybrid immunity compared to individuals with solely infection-induced immunity (14).

Considering the lack of specific data for the new vaccine proposed for the annual vaccination program, we used estimates of the bivalent mRNA vaccine’s effectiveness as a reference for the new vaccine. We assumed the values for vaccine effectiveness to be $\sigma_{B}=$ 0.29 (indicating a reduction in infection rate) and $\omega_{B}=$ 0.62 (signifying a decrease in the likelihood of hospitalization), grounded on the effectiveness study of bivalent boosters against Omicron subvariants (15). These assumptions about the effectiveness of immunity against infection and severe illness were integrated into our model to evaluate the implications of different vaccination strategies.

### 2.4 Scenario design

In this study, we conducted simulations to assess the impact of an annual vaccination program using various vaccine distribution strategies vs. no additional vaccination in South Korea. We stipulated that individuals over 6 months old would qualify for the new vaccination initiative, in line with South Korea’s existing annual COVID-19 vaccination program plan (2). We set a baseline annual attack rate—the cumulative proportion of individuals who become infected—of 20% for the 1-year period. Additionally, we also considered a higher attack rate (30%) in case a new variant with increased transmissibility emerges. For both the 20% and 30% attack rate scenarios, we assumed the program would run from October 1, 2023 to December 31, 2023.

COVID-19 vaccine uptake in South Korea has varied among different campaigns. Earlier primary series campaigns achieved high uptake levels of 94%, but this rate dropped to 15% during the winter 2022 campaign (3). To accommodate this variability, we employed a conservative assumption of 20% vaccine uptake for our baseline analysis. We have included results for a scenario that assumes a higher uptake level of 30% for sensitivity analysis (Supplementary material).

We simulated three different allocation scenarios for eligible age groups (Table 1). In all scenarios assuming a baseline attack rate of 20%, we assumed that 10,257,991 vaccine doses would be administered, achieving a 20% vaccine coverage among the vaccine-eligible population. The first scenario entailed a uniform vaccine distribution, without a specific prioritization strategy, setting the vaccine uptake levels for each eligible age group uniformly at 20%. For prioritization strategies, we considered two scenarios: the second scenario targeted those aged ≥65 years, while the third scenario expanded the priority age group to those aged ≥50 years. We assumed vaccination of the prioritization group would commence for the first 30 days and then extend to all eligible age groups. For the second scenario, the vaccine uptake level for those aged ≥65 years was set at 40%. In the third scenario, for those aged ≥50 years, the vaccine uptake level was set at 30%. These vaccine uptake levels for priority groups were based on the observed coverage levels within each age group during the winter 2022 COVID-19 vaccination program (3). After allocating vaccines to these priority age groups, the remaining doses were distributed evenly among the non-priority age groups. For the sensitivity analysis, assuming a higher vaccine uptake level scenario (i.e., 30%), we hypothesized that 15,386,986 vaccine doses would be administered, achieving 30% vaccine coverage among the eligible population (Supplementary Table S2). We then set the vaccine uptake levels to 50% for prioritizing those aged 65 years and older, and to 40% for another scenario focusing on those aged 50 years and older. The resulting numbers of vaccinated individuals and vaccine uptake levels by age group for each vaccination strategy are displayed in Table 1 and Supplementary Table S2.

**Table 1:** Description of scenario analyses with 20% vaccine uptake level.

	Vaccination strategy	Number of vaccinated individuals among various age groups, n (proportion of age groups, %)			
		0.5–49 years	50–64 years	65+ years	All eligible group (0.5+ years)
Scenario 1	Uniform vaccine allocation	5,795,386 (20.0)	2,576,021 (20.0)	1,886,584 (20.0)	10,257,991 (20.0)
Scenario 2	Prioritization for individuals aged ≥65	4,489,336 (15.5)	1,995,488 (15.5)	3,773,168 (40.0)	
Scenario 3	Prioritization for individuals aged ≥50	3,564,084 (12.3)	3,864,031 (30.0)	2,829,876 (30.0)	

We considered three different allocation scenarios: (1) uniform vaccine distribution, without a specific prioritization strategy; (2) prioritization for individuals aged ≥65 years; and (3) expanding the priority age group to those aged ≥50 years. The vaccine uptake level for priority groups in the second scenario was set at 40% for those aged ≥65 years, while in the third scenario, the vaccine uptake level for those aged ≥50 years was set at 30%. After allocating vaccines to these priority age groups, the remaining doses were distributed evenly among the non-priority age groups.

### 2.5 COVID-19-related costs

Our analysis accounted for various direct and indirect costs associated with COVID-19 patients and the vaccination program. All costs were initially adjusted for inflation using the consumer price index, and then converted into US dollars by using the average exchange rate for June 2023 (16, 17).

We included direct costs linked to the medical care of symptomatic COVID-19 patients, encompassing expenditures related to hospitalization or outpatient care such as general physician visits. We used an average daily treatment cost of US$ 557.3 for COVID-19-related hospitalization (18, 19). For outpatient care costs, we employed the average home care costs per COVID-19 patient, estimated to be US$ 50.5 (18, 20). The standard cost for a dose of the COVID-19 vaccine was set at US$ 28.4, reflecting the average price per dose for bivalent vaccines (21). We also factored in an administration fee of US$ 15.1, as regulated by the Korea Disease Control and Prevention Agency (KDCA) (22).

Indirect costs in our calculations from a societal perspective considered productivity losses due to severe illness and death from COVID-19. The impact on lost workdays was evaluated using the average gross domestic product (GDP) *per capita* in Korea, US$ 34,998 *per capita*, along with the duration of hospitalization and outpatient visits (23). Furthermore, we calculated the economic effect of COVID-19-related mortality based on the years of life lost and an average productivity measure.

### 2.6 Loss of QALYs

In our investigation, we quantified the QALYs lost attributable to COVID-19, a metric which integrates both the quantity and quality of life lived. We took into account the diminished quality associated with symptomatic periods or during hospital interventions but posited no QALY decrement for asymptomatic cases (Table 2).

**Table 2:** Health states and associated disability weights used for determining quality-of-life weights.

Health state	Description	Disability weight (95% UI)	References
Infectious without symptoms	Infected but does not experience any symptoms	0	Assumed
Infectious with symptoms	Having a fever and aches, and feeling weak, which makes some difficulty to carry out daily activities	0.05 (0.03–0.07)	(24)
Hospitalized	Hospitalized in intensive care unit admission; or having a high fever and pain, and feeling very weak, which makes great difficulty to carry out daily activities	0.19 (0.14–0.24)	(24, 25)

Quality-adjusted life year decrements for symptomatic cases were derived from disutility weights, as detailed in the 2013 Global Burden of Disease study, reflecting the diminished health quality during moderate acute infectious disease episodes (24, 25). This study derived disutility weights from web-based survey data, using paired comparison questions in which respondents evaluated two hypothetical individuals’ health states and specified the healthier person. Values for quality-of-life weights were determined by subtracting disability weights from one. Patients undergoing clinical interventions for COVID-19 were allocated a quality-of-life weight of 0.95, with a 95% uncertainty interval [UI] of 0.93–0.97 (24). Those necessitating hospitalization received a weight of 0.81 (95% UI: 0.75–0.86), mirroring disability weights pertinent to intensive episodes and ICU admissions (24, 25). In our analysis, these values were applied on a daily basis, corresponding to symptomatic durations and hospitalizations. We assumed that COVID-19 related hospital stays averaged at 11 days, while non-hospitalized patients exhibited symptoms for approximately 7 days. To ascertain the number of life years lost due to COVID-19-related mortality, we used age-specific standard life expectancy data along with a discount rate of 4.5% per year (26). This framework enabled us to quantify the impact of COVID-19 on both the duration and quality-of-life experienced by those afflicted by the disease.

### 2.7 Cost-effectiveness analysis

We conducted a cost-effectiveness analysis of the annual COVID-19 vaccination program by considering the balance between the costs associated with vaccination, the decrease in medical expenses due to a reduced number of infections, and the incremental health benefits that could be achieved through the program.

We calculated the incremental costs and incremental QALYs associated with different annual vaccination strategies in comparison to a scenario without additional vaccination. The incremental cost-effectiveness ratio (ICER), which represents the extra cost per QALY gained, was computed from both healthcare and societal perspectives.

In this analysis, a vaccination strategy was deemed cost-effective if the cost per QALY gained was less than US$ 34,998 (23). This threshold was determined based on South Korea’s GDP *per capita*, a benchmark that is commonly used in cost-effectiveness analyses.

Given the inherent uncertainty in such analyses, we conducted a probabilistic sensitivity analysis to evaluate the robustness of our results. We performed 3,000 simulations to assess the likelihood of the vaccination program being deemed cost-effective under different cost-effectiveness thresholds. We also identified the cost per dose and effectiveness thresholds at which the vaccination program would be considered cost-effective. All the economic parameters along with their baseline values and ranges used in this analysis are detailed in Supplementary Table S1.

## 3 Results

### 3.1 Projected disease burden in the absence of COVID-19 annual vaccination program in South Korea

The projected impact of COVID-19 in South Korea in the absence of a vaccination program, as depicted in Table 3, suggests a significant burden. Under an assumed outbreak attack rate of 20%, our model predicts 7.0 million symptomatic infections, 124,118 hospitalizations, and 4,576 fatalities within a single year. Notably, over half of all hospitalizations (55.2%) and the majority of deaths (87.8%) were predicted to occur among individuals aged 65 and older. The direct medical expenses associated with hospitalizations and outpatient treatments for COVID-19 were projected to reach around US$ 1.1 billion.

**Table 3:** Cases, disease burden, and cost-effectiveness of various vaccination strategies for COVID-19 outbreak over a one-year period in South Korea.

	Health outcomes						Economic outcomes							
	Symptomatic infection		Hospitalization		Death			Total cost (million US$)		Change in cost (million US$)			ICER (US$/QALY)	
	Total new symptomatic infection	Total new symptomatic infection prevented	Total new hospitalization	Total new hospitalization prevented	Total COVID-19 related death	Total COVID-19 related death prevented	Direct medical costs (million US$)	Healthcare perspective	Societal perspective	Healthcare perspective	Societal perspective	QALYs gained	Healthcare perspective	Societal perspective
Attack rate = 20%														
No vaccination	6,990,648	-	124,118	-	4,576	-	1,107	1,107	3,911	-	-	-	-	-
Uniform vaccine allocation	5,915,119	1,075,529	100,363	23,755	3,708	868	909	1,355	3,662	248	−249	9,616	25,787	CS
Prioritization for individuals aged ≥65	6,209,338	781,310	101,331	22,787	3,614	961	929	1,376	3,696	269	−215	10,043	26,754	CS
Prioritization for individuals aged ≥50	6,243,910	746,738	102,470	21,648	3,739	837	938	1,385	3,750	277	−161	8,991	30,851	CS
Attack rate = 30%														
No vaccination	10,489,643	-	186,617	-	6,937	-	1,664	1,664	5,883	-	-	-	-	-
Uniform vaccine allocation	9,197,808	1,291,835	154,815	31,802	5,743	1,194	1,405	1,852	5,417	188	−466	13,098	14,364	CS
Prioritization for individuals aged ≥65	9,551,106	938,537	154,326	32,290	5,506	1,431	1,420	1,867	5,404	203	−479	14,726	13,785	CS
Prioritization for individuals aged ≥50	9,584,987	904,656	155,917	30,700	5,708	1,229	1,432	1,878	5,484	214	−399	13,051	16,426	CS

^*^QALY, Quality-adjusted life year; ICER, Incremental cost-effectiveness ratio; CS, Cost-saving.

In a more severe scenario, with an increased attack rate of 30%, the disease burden escalates. Here, the model forecasts 10.5 million symptomatic infections, 186,617 hospitalizations, and 6,937 deaths within the same annual period if no vaccination program is implemented. From both the healthcare and societal perspectives, the total costs associated with these outcomes were estimated to be US$ 1.7 billion and 5.9 billion, respectively.

These projections underscore the substantial epidemiological and economic costs of COVID-19 and serve as a baseline for evaluating the potential benefits and cost-effectiveness of implementing a vaccination program.

### 3.2 Clinical and economic benefits of annual vaccination programs in South Korea

Our results underscore the vital role of yearly COVID-19 vaccination programs in alleviating disease burden, as evidenced in Table 3. Of the three allocation strategies considered, the uniform vaccine allocation appeared most effective at diminishing the number of symptomatic infections and hospitalizations, while prioritization for individuals aged 65 or older proved the most successful at reducing deaths. Compared to a no-vaccination scenario, distributing vaccines evenly across all eligible age groups could potentially decrease symptomatic infection cases and hospitalizations by 1.1 million and 23,755, respectively. Although such a strategy would incur an additional vaccination cost of around US$ 446.7 million, it may result in US$ 198.7 million savings in direct medical costs. From a healthcare perspective, the ICER associated with this strategy was the lowest of the three at US$ 25,787 per QALY, notably under the GDP *per capita* threshold, and was deemed cost-saving from a societal perspective.

By contrast, prioritizing vaccination for individuals aged 65 and above could prevent 781,310 symptomatic infections, 22,787 hospitalizations, and 961 deaths, thus saving US$ 177.9 million in direct medical expenses compared to a no-vaccination scenario. This strategy could generate 10,043 QALYs, with most (89.4%) attributed to preventing COVID-19 related fatalities. From a healthcare perspective, the ICER for this strategy is US$ 26,754/QALY and is cost-saving from a societal perspective.

Broadening the priority group to include individuals aged over 50 may reduce symptomatic infections, hospitalizations, and deaths by 746,738, 21,648, and 837, respectively, when compared to no vaccination. However, this expansion could potentially result in an increase in the disease burden compared to the other two strategies, namely, the uniform vaccination strategy and the one prioritizing those aged 65 and above. This outcome may be attributed to the lower vaccine uptake levels among the younger population, whose contact rates are higher than older adults (Table 1). Despite being cost-effective with an ICER of US$ 30,851/QALY, this strategy results in higher total costs and fewer QALY gains compared to prioritizing those aged 65 and above, making it a dominated strategy.

In summary, from a healthcare perspective, the most cost-effective approach was the uniform vaccination strategy, which did not prioritize any age group. This was closely followed by the strategy that prioritized vaccination for individuals aged 65 and older, and lastly by the strategy that extended priority to those aged 50 and above. Nevertheless, when looking from a societal perspective, each of these strategies was found to be cost-saving, highlighting the overall economic and health benefits of implementing a COVID-19 vaccination program.

### 3.3 Sensitivity analysis by varying attack rate and vaccine uptake level

In a scenario of a swift outbreak with a 30% attack rate (as detailed in Table 3), the value of vaccination is markedly highlighted, though the results differ from those under the baseline attack rate. Under these circumstances, the uniform vaccine allocation strategy could potentially prevent 1.3 million symptomatic infections, along with 31,802 hospitalizations, and 1,194 deaths. This strategy would result in total cost savings, including both direct and indirect costs, of roughly US$ 466 million and an additional 13,098 QALYs. From a healthcare perspective, the strategy proves cost-effective with an ICER of US$ 14,364/QALY, and from a societal perspective, it is deemed cost-saving.

When prioritizing vaccinations for individuals aged 65 or older, this strategy is projected to prevent 32,290 hospitalizations and 1,431 deaths, translating to a substantial savings of US$ 479 million in total costs from societal perspective. Furthermore, it could contribute an additional 14,726 QALYs. Interestingly, evaluated from a healthcare perspective, this approach result in a lower ICER of US$ 13,785/QALY, positioning it as the most cost-effective strategy among the ones analyzed. This strategy also qualifies as cost-saving when considered from a societal perspective.

If the priority group were expanded to include those aged over 50, predictions indicate that this approach could prevent 904,656 symptomatic infections, reduce hospitalizations by 30,700, and avert 1,229 deaths. This could result in approximately US$ 399 million in cost savings including both direct and indirect costs, along with an additional 13,051 QALYs. From a healthcare perspective, this strategy is deemed cost-effective, although it presents the highest ICER among the three strategies evaluated. Nevertheless, from a societal perspective, it is considered cost-saving.

In scenarios of higher vaccine uptake levels, it is projected that the disease burden would be more significantly reduced compared to scenarios with 20% uptake levels (Supplementary Table S5). Upon cost-effectiveness analysis, however, a similar pattern emerges to that observed with baseline parameters. The most cost-effective strategy from a healthcare perspective remains the uniform vaccine distribution, followed by prioritizing individuals aged 65 and older, and finally by the strategy extending priority to those aged 50 and above, regardless of the attack rates we considered. From a societal perspective, all vaccination scenarios prove cost-saving and cost-effective from a healthcare perspective, as their ICERs fall below the threshold for cost-effectiveness. These findings highlight the critical need to understand the virus’s transmissibility and the anticipated disease burden when selecting a vaccine distribution strategy.

### 3.4 Probabilistic sensitivity analyses

**Figure 2:**
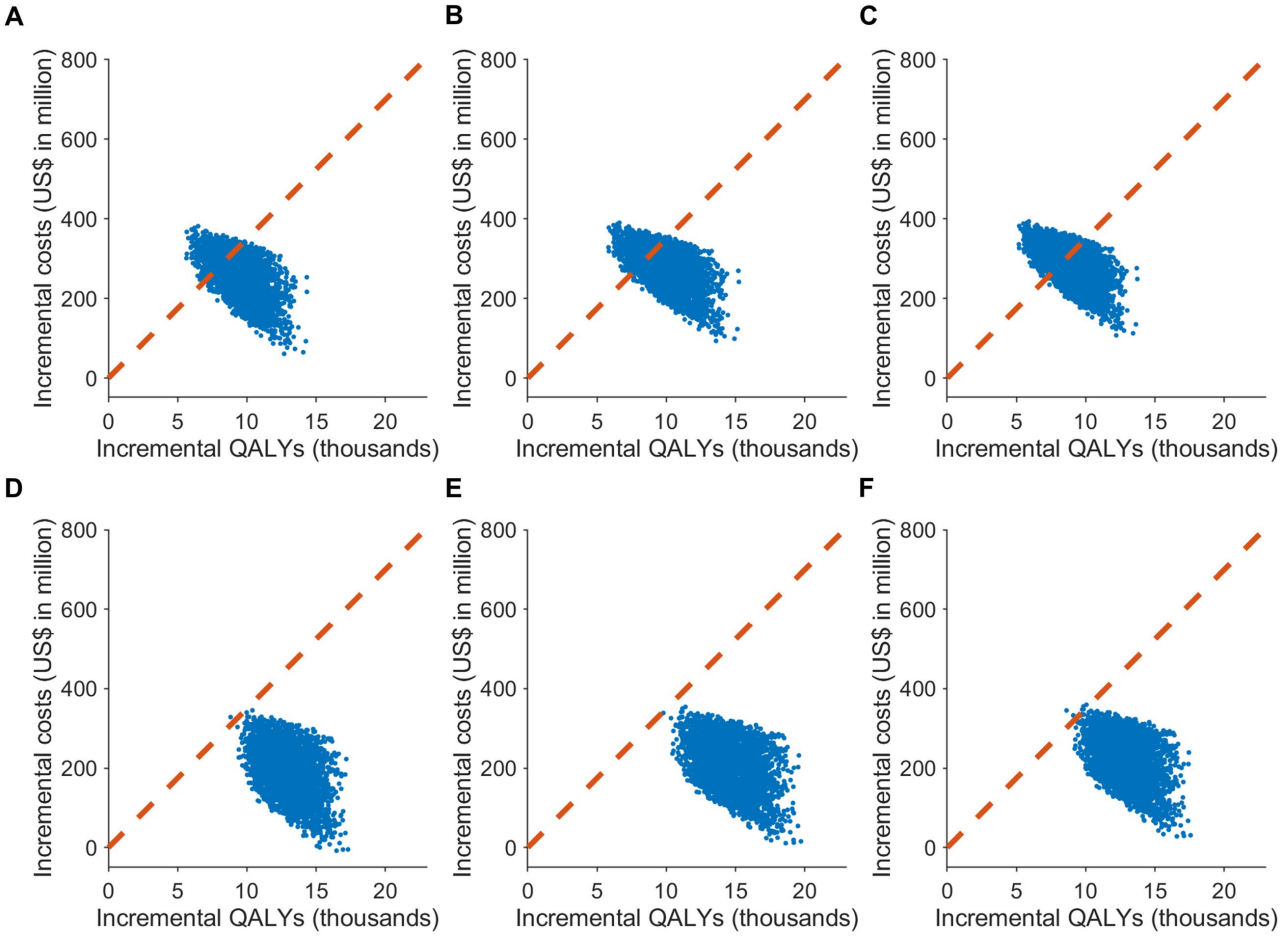
The cost-effectiveness plane of different COVID-19 vaccine allocation strategies compared to no vaccination in South Korea. It showcases the results of Monte Carlo simulations with 3,000 iterations, based on the value ranges and distributions specified in Supplementary Table S1, under two attack rate scenarios: **(A–C)** 20% and **(D–F)** 30%. The plane plots the incremental effect against the incremental cost associated with vaccination strategies, relative to no vaccination. The scenarios include: **(A,D)** uniform vaccine distribution; **(B,E)** prioritization for individuals aged 65 or older; and **(C,F)** prioritization for individuals aged 50 or older. The cost-effectiveness plane displays the outcomes of the probabilistic sensitivity analysis, with the dotted line signifying the cost-effectiveness threshold of US$ 34,998/QALY.

**Figure 3:**
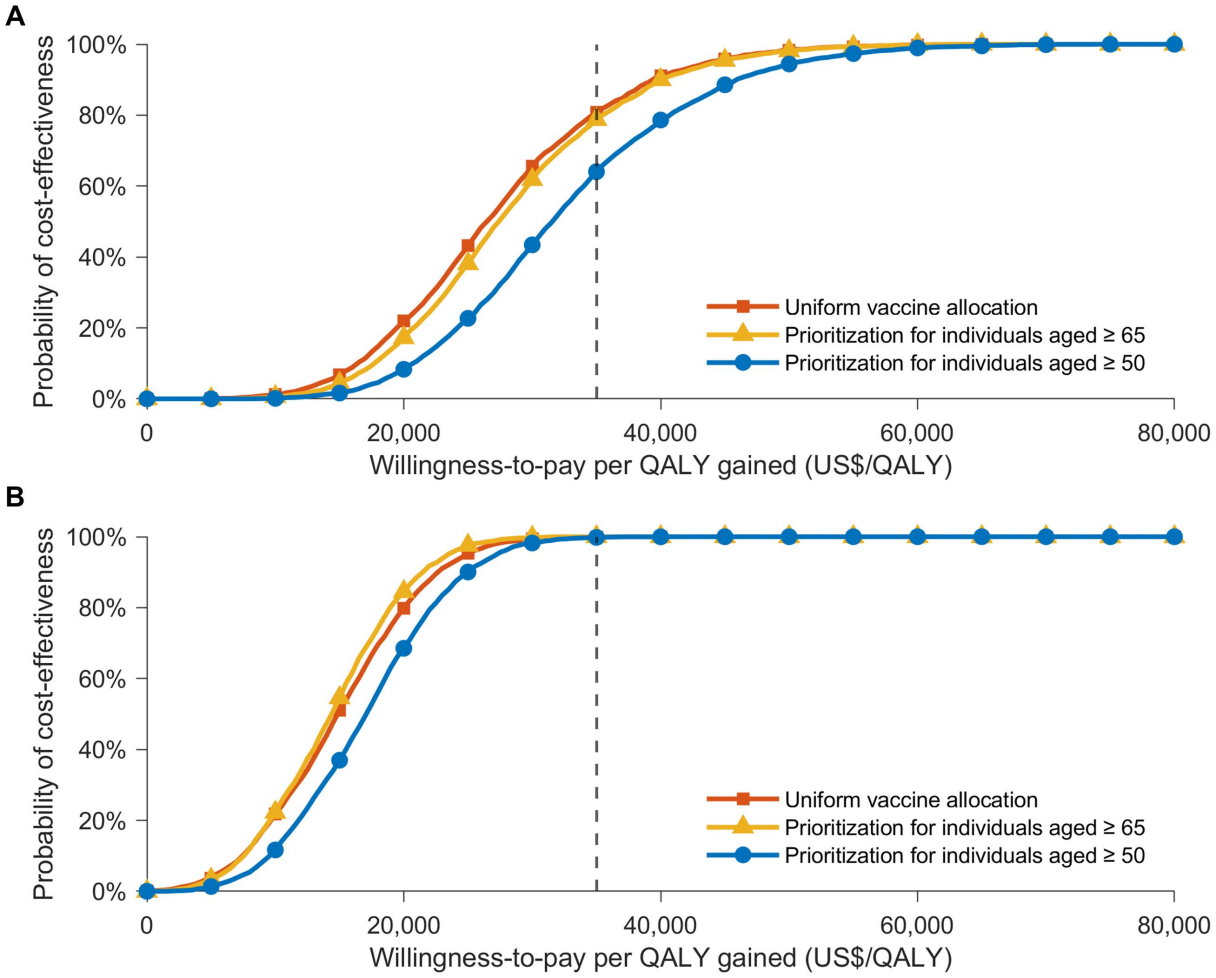
The cost-effectiveness acceptability curves showing the likelihood that various vaccine distribution strategies are cost-effective at distinct willingness-to-pay thresholds, in contrast to a no-vaccination strategy. These curves present the results of Monte Carlo simulations with 3,000 iterations, based on the value ranges and distributions detailed in Supplementary Table S1, under two infection rate scenarios: **(A)** 20% and **(B)** 30%. Acceptability curves are presented for three vaccine allocation strategies: uniform vaccine distribution (orange), prioritization for individuals aged 65 or older (yellow), and prioritization for those aged 50 or older (blue). A gray dotted line marks the cost-effectiveness threshold of US$ 34,998/QALY.

Probabilistic sensitivity analyses supplied deeper insights into potential outcome variations at different attack rates, influenced by various factors (Figures 2, 3). The cost-effectiveness acceptability curves, evaluating the probability of strategies being cost-effective across diverse willingness-to-pay thresholds, revealed that a uniform vaccination strategy had the highest likelihood of cost-effectiveness at the GDP *per capita* threshold of US$ 34,998/QALY, with a probability of 80.7% (Figure 3A). The strategies prioritizing vaccination for those aged 65 and over and those aged 50 and over also demonstrated high probabilities of cost-effectiveness, at 78.8% and 64.1%, respectively. However, in a severe COVID-19 outbreak scenario with a 30% attack rate, the strategy that prioritized individuals aged 65 and over had the highest likelihood of cost-effectiveness up to a threshold of US$ 30,000/QALY. Nevertheless, all vaccination allocation strategies displayed a 100% likelihood of cost-effectiveness compared to not vaccinating, at the GDP *per capita* threshold of US$ 34,998/QALY (Figure 3B).

**Figure 4:**
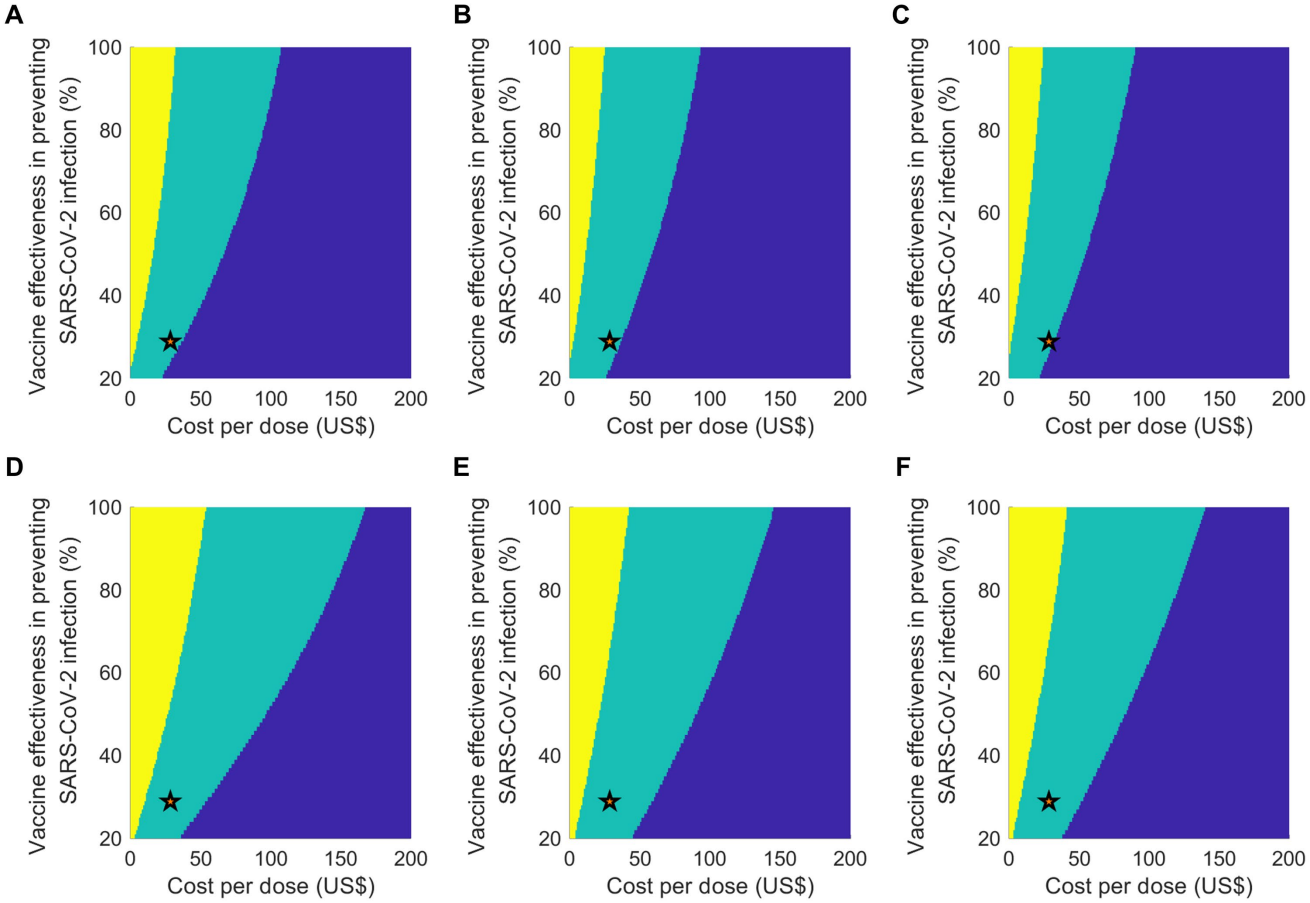
Outcomes of a two-way sensitivity analysis which varies vaccine efficacy against infection and cost per vaccinated person, considering two attack rate scenarios: **(A–C)** 20% and **(D–F)** 30%. The color-coded regions denote different cost-effectiveness results: yellow for cost-saving, green for cost-effective, and blue for not being cost-effective. The scenarios under analysis are: **(A,D)** equal vaccine distribution; **(B,E)** priority for individuals aged 65 or above; and **(C,F)** priority for individuals aged 50 or above. A star symbol is used to represent the baseline parameters.

The two-way sensitivity analysis offered insights into the cost-effectiveness of vaccination programs by varying both the vaccine effectiveness against infection and the vaccine cost per dose simultaneously (Figure 4). For a uniform vaccine allocation strategy with a 20% vaccine uptake level, assuming a vaccine effectiveness against infection of 29% (the baseline value), the maximum acceptable cost per dose was US$ 36. Regardless of the allocation strategies considered at a 20% attack rate, the vaccination program would not be cost-effective if the cost per dose surpassed US$ 107, even if the vaccine was hypothetically 100% effective. In a scenario with a higher attack rate of 30%, the maximum acceptable cost per dose increased, particularly for a uniform vaccine allocation strategy, with the limit reaching US$ 167 per dose. We also noted that the cost-effectiveness of the programs was more sensitive to changes in the cost per dose than changes in vaccine effectiveness. These findings underscore the significant influence of both vaccine efficacy and cost on the cost-effectiveness of COVID-19 vaccination programs.

## 4 Discussion

This study evaluated the epidemiological and economic impact of implementing an annual COVID-19 vaccination program in South Korea, using age-structured mathematical modeling. The findings reveal that an annual vaccination program would significantly reduce the burden of COVID-19-related diseases by decreasing the number of symptomatic infections, hospitalizations, and deaths. The most effective method for decreasing the number of infections would be a vaccination strategy without age-specific prioritization. Conversely, a strategy that prioritizes older adults, who are more susceptible to severe disease, yielded the greatest reduction in fatalities. This aligns with previous studies emphasizing the need to prioritize older adults to minimize mortality (27–29). Overall, the annual COVID-19 vaccination program was found to be highly cost-effective, which is consistent with earlier cost-effectiveness analyses of COVID-19 vaccines (7, 30, 31).

In South Korea, the number of confirmed cases has been increasing rapidly since late June 2023 (1). However, a South Korean survey regarding the willingness to participate in an annual COVID-19 vaccination program found that 8.4% of respondents would definitely not get the vaccine, while 24.7% probably would not (32). This suggests that vaccine hesitancy persists in South Korea, implying that the vaccine uptake level under an annual vaccination program might be relatively low. Significantly, our study indicates that a vaccination strategy with uniform distribution across age groups is more cost-effective than a strategy that prioritizes older adults, given a 20% attack rate. In a scenario of a severe outbreak, however, preference shifts toward a strategy prioritizing individuals aged 65 and above. Interestingly, as vaccine uptake level increases, a uniform vaccine allocation strategy consistently outperforms prioritizing strategies in terms of cost-effectiveness, irrespective of the attack rate. Our findings, therefore, suggest that both the disease burden and the level of vaccine uptake can influence the selection of age groups to prioritize when formulating vaccination strategies.

Potential financial challenges could emerge as vaccine manufacturers, such as Pfizer and Moderna, suggest possible increases in the price of bivalent booster doses (21, 33). This presents a significant financial burden for countries like South Korea, which rely heavily on imported vaccines (3, 33). Our analysis showed that the vaccination program would not be cost-effective from a healthcare perspective if the cost per vaccine dose exceeded US$ 107, even when assuming maximum effectiveness against infection. At the same vaccine effectiveness as the bivalent vaccine (29%), the highest cost per dose that would make an annual vaccination program cost-effective was found to be US$ 36 for a uniform vaccine allocation strategy.

The study has some limitations, such as assumptions of uniform vaccine effectiveness across all age groups, absence of additional risk group stratification, and an inability to accurately predict future outbreaks and emerging variants. Moreover, outcomes may be influenced by the assumed participation rate of vaccine-eligible individuals, as well as the timing of both the vaccination program and the outbreak peak.

In conclusion, this study highlights the potential health benefits and cost-effectiveness of implementing an annual COVID-19 vaccination program in South Korea. The findings suggest a potential shift in current vaccination strategies, with a universal vaccination program being more cost-effective compared to a strategy primarily focused on prioritizing older adults. Conversely, if a rapid and contagious outbreak is expected in South Korea during winter 2023, alongside continued vaccine hesitancy, prioritizing individuals aged 65 and older could be the most cost-effective option. By quantifying the costs and benefits of different vaccination strategies, this study provides valuable insights to guide policy-making and resource allocation decisions in the ongoing battle against COVID-19.

## Data availability statement

The original contributions presented in the study are included in the article/Supplementary material, further inquiries can be directed to the corresponding author.

## Ethics statement

This study was exempt from ethical approval due to a waiver that was granted by the Institutional Review Board of Soongsil University. The studies were conducted in accordance with the local legislation and institutional requirements. Written informed consent for participation was not required from the participants or the participants’ legal guardians/next of kin in accordance with the national legislation and institutional requirements.

## Author contributions

WC: Formal analysis, Investigation, Methodology, Validation, Writing – original draft, Writing – review & editing, Data curation, Software, Visualization. ES: Formal analysis, Investigation, Methodology, Validation, Writing – original draft, Writing – review & editing, Conceptualization, Funding acquisition, Project administration, Resources, Supervision.
