# Supplementary material for: Assessing the cost-effectiveness of annual COVID-19 booster vaccination in South Korea using a transmission dynamic model
Source: Front Public Health. 2023 Nov 23;11:1280412. doi: 10.3389/fpubh.2023.1280412 (PMC10701673; doi:10.3389/fpubh.2023.1280412)
Supplement: Supplementary file 1 [file Data_Sheet_1.docx]

Supplementary Material

# Supplementary methods

## Model structure

We developed a deterministic compartmental model to simulate disease progression of severe acute respiratory syndrome coronavirus 2 (SARS-CoV-2) infection and annual vaccination program. This model extended the susceptible-vaccinated-exposed-asymptomatic-symptomatic-recovered (SVEAIR) model structure of SARS-CoV-2 transmission by incorporating age stratifications and pre-existing immunity against COVID-19.

The population was divided into 17 age groups for those individuals under five months, six months to four years, five to nine years, …, 70 to 74, and 75+ years. For the annual vaccination program, we considered three initial immune statuses: fully susceptible, partially susceptible, and the immunized group under the annual vaccination program. Individuals who had both infection- and vaccination-induced antibodies, denoted as having hybrid immunity, were assumed to have greater immunity compared to those individuals who were fully susceptible. The grouping was based on the evidence indicating that hybrid immunity provided stronger and longer-lasting protection against infection (1-5).

After SARS-CoV-2 infection and regardless of immune status, an individual may progress through the following health states.

- Latent: infected but not yet infectious.
- Infectious without symptoms: infectious individuals who do not develop symptoms at any stage of the disease course.
- Infectious with symptoms: infectious individuals who experience symptoms and who would be reported and counted as outpatients.
- Hospitalized: infected individuals who develop severe symptoms, which is managed by isolation from the rest of the patient population at a hospital.
- Recovered from asymptomatic infection: individuals who recover from asymptomatic infection and/or may be unaware of infection.
- Recovered from symptomatic infection: individuals who recover from symptomatic infection and recognize their infection.

Since the model was simulated over a one-year period, we disregarded natural births and deaths.

We considered that individuals over the age of six months would be eligible for vaccination based on the current plan of the annual vaccination program in South Korea (5). Vaccination was assumed to occur at an age-specific rate in our model through the annual vaccination program for individuals who were susceptible, exposed, asymptomatic, or naturally recovered from the virus. However, only susceptible individuals could benefit from the protective effectiveness of vaccination. Symptomatic individuals or those with confirmed infection were not considered for vaccination.

## Model equations

The state variables are as follows: fully susceptible ($S_{k}$), partially susceptible due to hybrid immunity ($Z_{k}$), immunized with annual vaccination $\left( B_{k} \right)$, latent $\left( E_{k}^{x} \right)$, infectious without symptoms $\left( A_{k}^{x} \right)$, infectious with symptoms $\left( I_{k}^{x} \right)$, hospitalized $\left( J_{k}^{x} \right)$, recovered from asymptomatic infection $\left( R_{k}^{x} \right)$, and recovered from symptomatic infection $\left( G_{k}^{x} \right)$, where the subscript $k$ refers to the age group ($k=1, \ldots, 17$) and superscript $x$ refers to the immune status ($x=S, Z, B).$ We presented the model equations for each of the immune status.

### Force of infection for transmission model

The time- and age-dependent force of infection, $\lambda_{k}\left( t \right)$, represented the rate at which susceptible individuals in age group $k$ became infected:

$$\lambda_{k}\left( t \right)=\beta_{k}\sum_{l=1}^{17} \phi_{kl}\frac{\sum_{x\in\left\{ S, Z, B \right\}} \left( A_{l}^{x}\left( t \right)+I_{l}^{x}\left( t \right) \right)}{N_{l}-\sum_{x\in\left\{ S, Z, B \right\}} \left( J_{l}^{x}\left( t \right) \right)}.$$

It was computed as the product of age-specific probability $\beta_{k}$ of infection after contact with an infectious individual, the contact patterns $\phi_{kl}$, which determined the number of contacts an age-$k$ individual had with age-$l$ individuals per day, and the proportion of infectious individuals (asymptomatic and symptomatic) in each age group, adjusted for the number of individuals already hospitalized. Here, the number of individuals in the age group $k$ is denoted by $N_{k}$. The contact matrix for South Korea estimated by Prem et al. was used (6).

### Fully susceptible individuals

Upon infection at the rate of time- and age-dependent force of infection, $\lambda_{k}\left( t \right)$, the average duration of the latency period ($1/\epsilon$) was assumed to be 3.1 days, regardless of the immune status (7-9). The probability of developing symptomatic disease requiring medical attention or hospitalization, denoted as $p_{k}$, varied by age. The average infectious period for individuals was assumed to be seven days ($1/\gamma=7$), and all infected individuals were assumed to have equal infectiousness regardless of symptom severity (10-15). A proportion, $h_{k}/(\gamma+h_{k})$, of infected individuals with symptoms in age group $k$ were assumed to be hospitalized. Hospitalized individuals either recovered after an average of 11 days ($1/\gamma_{J}=11$) or died from COVID-19 at an age-specific rate $\mu_{k}$ (16, 17). Individuals who recovered from infection were considered fully immune to COVID-19 for the remaining duration of the outbreak. The epidemiological dynamics for fully susceptible individuals are described by the following system of differential equations:

$$\frac{dS_{k}\left( t \right)}{dt}=-\lambda_{k}\left( t \right)S_{k}\left( t \right)-\psi_{k}\left( t \right)S_{k}\left( t \right)$$

$$\frac{dE_{k}^{S}\left( t \right)}{dt}=\lambda_{k}\left( t \right)S_{k}\left( t \right)-\epsilon E_{k}^{S}(t)$$

$$\frac{dA_{k}^{S}\left( t \right)}{dt}=\epsilon\left( 1-p_{k} \right)E_{k}^{S}\left( t \right)-\gamma A_{k}^{S}\left( t \right)$$

$$\frac{dI_{k}^{S}\left( t \right)}{dt}=\epsilon p_{k}E_{k}^{S}\left( t \right)-\left( h_{k}+\gamma\right)I_{k}^{S}\left( t \right)$$

$$\frac{dJ_{k}^{S}\left( t \right)}{dt}=h_{k}I_{k}^{S}\left( t \right)-\left( \gamma_{J}+\mu_{k} \right)J_{k}^{S}\left( t \right)$$

$$\frac{dR_{k}^{S}\left( t \right)}{dt}=\gamma A_{k}^{S}\left( t \right)$$

$$\frac{dG_{k}^{S}\left( t \right)}{dt}=\gamma I_{k}^{S}\left( t \right)+\gamma_{J}J_{k}^{S}\left( t \right)$$

### Partially susceptible individuals

Individuals with partial immunity resulting in hybrid immunity were assumed to have reduced likelihood of infection per infectious contact and severe disease following infection. We denoted a reduction in the infection rate as $\sigma_{Z}$ and a decrease in the probability of severe illness requiring hospitalization as $\omega_{Z}$, compared to fully susceptible individuals ($S$). The rate of hospitalization for partially susceptible individuals, $h_{k}^{Z}$, was determined by calculating the reduction in the proportion of hospitalizations attributed to protective effectiveness ($\omega_{Z}$), that is,

$$\frac{h_{k}^{Z}}{h_{k}^{Z}+\gamma}=\left( 1-\omega_{Z} \right)\frac{h_{k}}{h_{k}+\gamma}.$$

We assumed a protective effectiveness against infection ($\sigma_{Z}$) of 0.15. The assumption of a linear decrease in protection conferred by hybrid immunity with primary doses informed this value, given the estimates of hybrid immunity effectiveness against reinfection at three months [69.0% with 95% confidence interval (CI): 58.9 to 77.5) and 12 months (41.8% with 95% CI: 31.5 to 52.8] (1). Similarly, the protective effectiveness against hospitalization $\left( \omega_{Z} \right)$ among partially susceptible individuals was assumed to be 0.31. This value was calculated based on the relative risk of hospitalization in individuals with hybrid immunity and primary series vaccination compared to those with previous infection in the absence of vaccination (1). The epidemiological dynamics for partially susceptible individuals are described by the following system of differential equations:

$$\frac{dZ_{k}\left( t \right)}{dt}=-\left( 1-\sigma_{Z} \right)\lambda_{k}\left( t \right)Z_{k}\left( t \right)-\psi_{k}\left( t \right)Z_{k}\left( t \right)$$

$$\frac{dE_{k}^{Z}\left( t \right)}{dt}=\left( 1-\sigma_{Z} \right)\lambda_{k}\left( t \right)Z_{k}\left( t \right)-\epsilon E_{k}^{Z}(t)$$

$$\frac{dA_{k}^{Z}\left( t \right)}{dt}=\epsilon\left( 1-p_{k} \right)E_{k}^{Z}\left( t \right)-\gamma A_{k}^{Z}\left( t \right)$$

$$\frac{dI_{k}^{Z}\left( t \right)}{dt}=\epsilon p_{k}E_{k}^{Z}\left( t \right)-\left( h_{k}^{Z}+\gamma\right)I_{k}^{Z}\left( t \right)$$

$$\frac{dJ_{k}^{Z}\left( t \right)}{dt}=h_{k}^{Z}I_{k}^{Z}\left( t \right)-\left( \gamma_{J}+\mu_{k} \right)J_{k}^{Z}\left( t \right)$$

$$\frac{dR_{k}^{Z}\left( t \right)}{dt}=\gamma A_{k}^{Z}\left( t \right)$$

$$\frac{dG_{k}^{Z}\left( t \right)}{dt}=\gamma I_{k}^{Z}\left( t \right)+\gamma_{J}J_{k}^{Z}\left( t \right)$$

### Vaccinated individuals through an annual vaccination program

Vaccination was assumed to occur at an age-specific rate of $\psi_{k}\left( t \right)$ through the annual vaccination program for individuals. Only susceptible individuals are assumed to benefit from the protective effectiveness of vaccination. Similar to the partially susceptible group, vaccinated individuals track the same epidemiological states as fully susceptible individuals but gain the added advantage from vaccination of a reduced likelihood of infection per infectious contact ($\sigma_{B}$) and reduced severity of disease after infection ($\omega_{B}$), compared to fully susceptible individuals ($S$). The rate of hospitalization among vaccinated individuals ($h_{k}^{B}$) was derived from $\frac{h_{k}^{B}}{h_{k}^{B}+\gamma}=\left( 1-\omega_{B} \right)\frac{h_{k}}{h_{k}+\gamma}$, considering the reduction in the proportion of hospitalizations attributed to protective effectiveness ($\omega_{B}$).

Due to the lack of specific data for the new vaccine proposed for the annual vaccination program at the time of initiation of our study, we used estimates of the bivalent mRNA vaccine effectiveness as a baseline for the new vaccine. The assumed values for vaccine effectiveness were $\sigma_{B}=$ 0.29 (reduction in infection rate) and $\omega_{B}=$ 0.62 (reduction in probability of hospitalization) (18). The epidemiological dynamics for immunized individuals with annual vaccination are described by the following system of differential equations:

$$\frac{dB_{k}\left( t \right)}{dt}=-\left( 1-\sigma_{B} \right)\lambda_{k}\left( t \right)B_{k}\left( t \right)+\psi_{k}\left( t \right)\left( S_{k}\left( t \right)+Z_{k}\left( t \right) \right)$$

$$\frac{dE_{k}^{B}\left( t \right)}{dt}=\left( 1-\sigma_{B} \right)\lambda_{k}\left( t \right)B_{k}\left( t \right)-\epsilon E_{k}^{B}(t)$$

$$\frac{dA_{k}^{B}\left( t \right)}{dt}=\epsilon\left( 1-p_{k} \right)E_{k}^{B}\left( t \right)-\gamma A_{k}^{B}\left( t \right)$$

$$\frac{dI_{k}^{B}\left( t \right)}{dt}=\epsilon p_{k}E_{k}^{B}\left( t \right)-\left( h_{k}^{B}+\gamma\right)I_{k}^{B}\left( t \right)$$

$$\frac{dJ_{k}^{B}\left( t \right)}{dt}=h_{k}^{B}I_{k}^{B}\left( t \right)-\left( \gamma_{J}+\mu_{k} \right)J_{k}^{B}\left( t \right)$$

$$\frac{dR_{k}^{B}\left( t \right)}{dt}=\gamma A_{k}^{B}\left( t \right)$$

$$\frac{dG_{k}^{B}\left( t \right)}{dt}=\gamma I_{k}^{B}\left( t \right)+\gamma_{J}J_{k}^{B}\left( t \right)$$

## Vaccination scenarios

A conservative assumption of a 20% vaccine uptake level was used as baseline to analyze three different allocation scenarios for eligible age groups: (1) uniform vaccine allocation without any specific prioritization, (2) prioritization for individuals aged 65 and above, and (3) prioritization for individuals aged 50 and above. For sensitivity analysis, a 30% vaccine uptake level was considered.

Regarding the prioritization strategy, we assumed that vaccination would initiate with the prioritization group within the first 30 days and then expand to include all eligible age groups. The program was anticipated to run from October 1, 2023, to December 31, 2023.

Mathematically, age-specific rates of $\psi_{k}\left( t \right)$ with each allocation scenario were defined as follows:

- For Scenario 1 $(k=2,\ldots,17$):

$$\psi_{k}\left( t \right)=\left[ \begin{matrix} 0 & t<October 1, 2023 \\ c_{k} & October 1, 2023\leq t\leq December 31, 2023 \\ 0 & December 31, 2023<t \end{matrix} \right.$$

- For priority groups in Scenario 2$(k=15, 16,\mathrm{and}17$) and Scenario 3$(k=12,\ldots,17$):

$$\psi_{k}\left( t \right)=\left[ \begin{matrix} 0 & t<October 1, 2023 \\ c_{k} & October 1, 2023\leq t<October 31, 2023 \\ c_{k} & October 31, 2023\leq t\leq December 31, 2023 \\ 0 & December 31, 2023<t \end{matrix} \right.$$

- For non-priority groups in Scenario 2$(k=2,\ldots,14$) and Scenario 3$(k=2,\ldots,11$):

$$\psi_{k}\left( t \right)=\left[ \begin{matrix} 0 & t<October 1, 2023 \\ 0 & October 1, 2023\leq t<October 31, 2023 \\ c_{k} & October 31, 2023\leq t\leq December 31, 2023 \\ 0 & December 31, 2023<t \end{matrix} \right.$$

Here, the variable $c_{k}$ is the age-specific vaccination rate derived from vaccine uptake level in each vaccination scenario.

# Supplementary Tables

**Supplementary Table S1. Description of epidemiological and economic parameters including baseline values and distribution.**

| **Model parameter** | **Value** | **Range^a^** | | **Ref** |
| --- | --- | --- | --- | --- |
|  |  | Low | High |  |
| **Epidemiological parameters** | | | | |
| Age-specific transmission probability, $\beta_{k}$ | Calibrated to attack rates |  |  | - |
| Number of age-$l$ individuals contacted by an age-$k$ individual per day, $\phi_{kl}$ | Supplementary Table S4 |  |  | (6) |
| Latent period (days), $1/\epsilon$ | 3.1 |  |  | (7-9) |
| Age-specific proportion of symptomatic infection, $p_{k}$ | 0.66 for $k=1-5$  0.74 for $k=6-9$  0.68 for $k=10-13$  0.62 for $k=14-17$ |  |  | (19-21) |
| Age-specific hospitalization ratio of symptomatic individuals (%), 100*$\frac{h_{k}}{h_{k}+\gamma}$ | 0.01 for $k=1-3$  0.33 for $k=4, 5$  0.50 for $k=6, 7$  0.70 for $k=8, 9$  0.96 for $k=10, 11$  1.98 for $k=12, 13$  3.65 for $k=14, 15$  11.02 for $k=16$  12.70 for $k=17$ |  |  | (22, 23) |
| Average infectious period (days), $1/\gamma$ | 7 |  |  | (10-12) |
| Average duration of hospitalization (days), $1/\gamma_{J}$ | 11 | 9 | 13 | (16) |
| Death rate of severe cases (%), 100*$\mu_{k}$ | 0 for $k=1, \ldots, 5$  0.0024 for $k=6, \ldots,9$  0.0341 for $k=10, \ldots,13$  0.2562 for $k=14$ and $15$  0.6256 for $k=16$ and $17$ |  |  | (17, 19) |
| **Relative reduction in the risk of infection compared to the fully susceptible individuals** | | | | |
| Partially susceptible group, $\sigma_{Z}$ | 0.15 | 0.07 | 0.22 | (1) |
| Individuals vaccinated through an annual vaccination program, $\sigma_{B}$ | 0.29 |  |  | (18) |
| **Relative reduction in the risk of hospitalization compared to the fully susceptible individuals** | | | | |
| Partially susceptible group, $\omega_{Z}$ | 0.30 | 0.15 | 0.45 | (1) |
| Individuals vaccinated through an annual vaccination program, $\omega_{B}$ | 0.62 |  |  | (18) |
| **Costs (US$)^b^** | | | | |
| Vaccine cost per dose | 28.42 |  |  | (24) |
| Vaccine administration cost | 15.12 |  |  | (25) |
| Medical cost for patients who need clinical treatments | 50.46 | 25.23 | 75.69 | (26, 27) |
| Medical cost for hospitalized patients per day | 557.28 | 278.64 | 835.92 | (27, 28) |
| **Quality of life weights** | | | | |
| For healthy population | 1 for $k=1, \ldots, 5$  0.97 for $k=6, \ldots,11$  0.96 for $k=12$ and $13$  0.94 for $k=14$  0.89 for $k=15, 16$ and $17$ |  |  | (29) |
| For patients who need clinical treatments | 0.95 | 0.93 | 0.97 | (30) |
| For patients who need hospitalization | 0.81 | 0.75 | 0.86 | (28, 30, 31) |
| **Discount rate (%)** | 4.5 |  |  | (32) |

^a^ For probabilistic sensitivity analysis, average duration of hospitalization, costs and quality of life weights were assigned uniform distributions, while vaccine efficacy estimates were assigned triangular distributions.

^b^ All costs were initially adjusted for inflation in the 2020 reference year using the consumer price index, and then converted into US dollars by using the average exchange rate for June 2023 (one US dollar = 1,296.7 Korean won) (33, 34).

**Supplementary Table S2.** Description of scenario analyses for a higher uptake level (30%). For sensitivity analysis, we considered a higher uptake level (30%) with three different allocation scenarios: (1) uniform vaccine distribution, without a specific prioritization strategy; (2) prioritization for individuals aged ≥65 years; and (3) expanding the priority age group to those aged ≥50 years. The vaccine uptake level for priority groups in the second scenario was set at 50% for those aged ≥65 years, while in the third scenario, the vaccine uptake level for those aged ≥50 years was set at 40%. After allocating vaccines to these priority age groups, the remaining doses were distributed evenly among the non-priority age groups.

|  | Vaccination strategy | Number of vaccinated individuals among various age groups, n (proportion of age groups, %) | | | |
| --- | --- | --- | --- | --- | --- |
|  |  | 0.5y-49y | 50y-64y | 65+y | All eligible group (0.5+y) |
| Scenario S1 | Uniform vaccine allocation | 8,693,079 (30.0) | 3,864,031 (30.0) | 2,829,876 (30.0) | 15,386,986 (30.0) |
| Scenario S2 | Prioritization for individuals aged ≥65 | 7,387,029 (25.5) | 3,283,498 (25.5) | 4,716,460 (50.0) |  |
| Scenario S3 | Prioritization for individuals aged ≥50 | 6,461,777 (22.3) | 5,152,042 (40.0) | 3,773,168 (40.0) |  |

**Supplementary Table S3.** Initial distribution of population.

| **Input description** | **0–9 years** | **10–19 years** | **20–29 years** | **30–39 years** | **40–49 years** | **50–59 years** | **60–69 years** | **70+ years** |
| --- | --- | --- | --- | --- | --- | --- | --- | --- |
| Proportion of fully susceptible individuals (%) | 99.1 | 48.2 | 29.7 | 30.3 | 29.8 | 34.3 | 35.6 | 46.2 |
| Proportion of partially susceptible individuals (%) ^a^ | 0.9 | 51.8 | 70.3 | 69.7 | 70.2 | 65.7 | 64.4 | 53.8 |

^a^ Proportion of partially susceptible individuals were based on the distribution of individuals who completed any two-dose primary series and N-positive proportion (5, 35).

**Supplementary Table S4. The contact matrix for South Korea (6).**

|  | **0–0.5** | **0.5–4** | **5–9** | **10–14** | **15–19** | **20–24** | **25–29** | **30–34** | **35–39** | **40–44** | **45–49** | **50–54** | **55–59** | **60–64** | **65–69** | **70–74** | **75+** |
| --- | --- | --- | --- | --- | --- | --- | --- | --- | --- | --- | --- | --- | --- | --- | --- | --- | --- |
| **0–0.5** | 0.15 | 1.62 | 0.82 | 0.30 | 0.20 | 0.26 | 0.41 | 0.61 | 0.61 | 0.32 | 0.19 | 0.22 | 0.18 | 0.14 | 0.11 | 0.07 | 0.04 |
| **0.5–4** | 0.15 | 1.62 | 0.82 | 0.30 | 0.20 | 0.26 | 0.41 | 0.61 | 0.61 | 0.32 | 0.19 | 0.22 | 0.18 | 0.14 | 0.11 | 0.07 | 0.04 |
| **5–9** | 0.07 | 0.81 | 8.43 | 1.05 | 0.24 | 0.16 | 0.31 | 0.51 | 0.69 | 0.60 | 0.27 | 0.16 | 0.15 | 0.15 | 0.11 | 0.06 | 0.04 |
| **10–14** | 0.02 | 0.19 | 2.29 | 11.67 | 0.81 | 0.25 | 0.20 | 0.29 | 0.49 | 0.68 | 0.41 | 0.20 | 0.11 | 0.09 | 0.09 | 0.07 | 0.06 |
| **15–19** | 0.01 | 0.13 | 0.34 | 3.81 | 11.77 | 0.89 | 0.38 | 0.27 | 0.41 | 0.57 | 0.63 | 0.31 | 0.12 | 0.08 | 0.06 | 0.04 | 0.03 |
| **20–24** | 0.02 | 0.26 | 0.23 | 0.28 | 2.65 | 3.65 | 1.49 | 0.93 | 0.77 | 0.73 | 0.97 | 0.69 | 0.37 | 0.18 | 0.08 | 0.08 | 0.06 |
| **25–29** | 0.05 | 0.56 | 0.26 | 0.14 | 0.47 | 1.64 | 3.20 | 1.77 | 1.34 | 1.14 | 1.00 | 0.98 | 0.55 | 0.29 | 0.11 | 0.05 | 0.03 |
| **30–34** | 0.07 | 0.72 | 0.68 | 0.46 | 0.24 | 0.76 | 1.59 | 2.55 | 1.69 | 1.30 | 1.07 | 0.86 | 0.66 | 0.36 | 0.16 | 0.07 | 0.06 |
| **35–39** | 0.08 | 0.83 | 1.18 | 0.85 | 0.42 | 0.57 | 1.29 | 1.67 | 2.84 | 1.92 | 1.29 | 0.98 | 0.60 | 0.43 | 0.25 | 0.14 | 0.06 |
| **40–44** | 0.05 | 0.50 | 1.00 | 1.24 | 0.81 | 0.78 | 1.20 | 1.61 | 1.84 | 2.79 | 1.73 | 1.21 | 0.53 | 0.41 | 0.23 | 0.14 | 0.07 |
| **45–49** | 0.03 | 0.31 | 0.67 | 0.90 | 1.20 | 0.93 | 1.08 | 1.29 | 1.50 | 1.69 | 2.33 | 1.35 | 0.69 | 0.37 | 0.19 | 0.15 | 0.13 |
| **50–54** | 0.05 | 0.51 | 0.57 | 0.79 | 0.99 | 1.17 | 1.54 | 1.37 | 1.30 | 1.75 | 2.01 | 2.15 | 1.18 | 0.55 | 0.22 | 0.15 | 0.12 |
| **55–59** | 0.07 | 0.81 | 0.89 | 0.59 | 0.75 | 0.89 | 1.50 | 1.66 | 1.27 | 1.42 | 1.27 | 1.60 | 1.86 | 0.88 | 0.36 | 0.17 | 0.12 |
| **60–64** | 0.09 | 1.00 | 1.00 | 0.64 | 0.66 | 0.70 | 1.18 | 1.38 | 1.43 | 1.28 | 1.13 | 1.10 | 1.32 | 1.46 | 0.63 | 0.37 | 0.15 |
| **65–69** | 0.06 | 0.61 | 0.94 | 0.72 | 0.40 | 0.47 | 0.73 | 0.98 | 0.87 | 0.90 | 0.58 | 0.68 | 0.70 | 0.70 | 0.94 | 0.33 | 0.16 |
| **70–74** | 0.03 | 0.31 | 0.88 | 0.68 | 0.62 | 0.22 | 0.42 | 0.40 | 0.68 | 0.74 | 0.56 | 0.46 | 0.37 | 0.67 | 0.63 | 0.82 | 0.29 |
| **75+** | 0.03 | 0.32 | 0.44 | 0.59 | 0.47 | 0.19 | 0.20 | 0.32 | 0.42 | 0.42 | 0.45 | 0.44 | 0.29 | 0.24 | 0.34 | 0.31 | 0.30 |

**Supplementary Table S5.** Cases, disease burden, and cost-effectiveness of various COVID-19 vaccination strategies with vaccine uptake level of 30% in South Korea.

|  | **Health outcomes** | | | | | | **Economic outcomes** | | | | | | | |
| --- | --- | --- | --- | --- | --- | --- | --- | --- | --- | --- | --- | --- | --- | --- |
|  | **Symptomatic infection, n** | | **Hospitalization, n** | | **Death, n** | |  | **Total cost (million US$)** | | **Change in cost (million US$)** | |  | **ICER (US$/QALY)** | |
|  | **Total new symptomatic infection** | **Total new symptomatic infection prevented** | **Total new hospitalization** | **Total new hospitalization prevented** | **Total COVID-19 related death** | **Total COVID-19 related death prevented** | **Direct medical costs (million US$)** | **Healthcare perspective** | **Societal perspective** | **Healthcare perspective** | **Societal perspective** | **QALYs gained** | **Healthcare perspective** | **Societal perspective** |
| **Attack rate = 20%** | | | | | | | | | | | | | | |
| No vaccination | 6,990,648 | - | 124,118 | - | 4,576 | - | 1,107 | 1,107 | 3,911 | - | - | - | - | - |
| Uniform vaccine allocation | 5,471,340 | 1,519,308 | 91,006 | 33,112 | 3,366 | 1,210 | 829 | 1,499 | 3,607 | 392 | -304 | 13,435 | 29,180 | CS |
| Prioritization for individuals aged ≥65 | 5,814,918 | 1,175,730 | 92,966 | 31,152 | 3,313 | 1,263 | 859 | 1,529 | 3,673 | 421 | -238 | 13,416 | 31,402 | CS |
| Prioritization for individuals aged ≥50 | 5,832,586 | 1,158,061 | 93,652 | 30,465 | 3,418 | 1,158 | 864 | 1,534 | 3,713 | 426 | -198 | 12,563 | 33,936 | CS |
| **Attack rate = 30%** | | | | | | | | | | | | | | |
| No vaccination | 10,489,643 | - | 186,617 | - | 6,937 | - | 1,664 | 1,664 | 5,883 | - | - | - | - | - |
| Uniform vaccine allocation | 8,603,428 | 1,886,214 | 141,000 | 45,617 | 5,226 | 1,710 | 1,291 | 1,961 | 5,238 | 298 | -645 | 18,810 | 15,815 | CS |
| Prioritization for individuals aged ≥65 | 9,045,046 | 1,444,596 | 142,237 | 44,379 | 5,053 | 1,883 | 1,321 | 1,991 | 5,278 | 327 | -605 | 19,711 | 16,605 | CS |
| Prioritization for individuals aged ≥50 | 9,060,051 | 1,429,592 | 143,216 | 43,401 | 5,232 | 1,705 | 1,328 | 1,998 | 5,341 | 334 | -542 | 18,288 | 18,263 | CS |

* Abbreviation: QALY, quality-adjusted life year; ICER, incremental cost-effectiveness ratio; CS, cost-saving.

**References**

1. Bobrovitz N, Ware H, Ma X, Li Z, Hosseini R, Cao C, et al. Protective effectiveness of previous SARS-CoV-2 infection and hybrid immunity against the omicron variant and severe disease: a systematic review and meta-regression. Lancet Infect Dis. 2023.

2. Woodbridge Y, Amit S, Huppert A, Kopelman NM. Viral load dynamics of SARS-CoV-2 Delta and Omicron variants following multiple vaccine doses and previous infection. Nat Commun. 2022;13(1):6706.

3. Levine-Tiefenbrun M, Yelin I, Alapi H, Herzel E, Kuint J, Chodick G, et al. Waning of SARS-CoV-2 booster viral-load reduction effectiveness. Nat Commun. 2022;13(1):1237.

4. Levin EG, Lustig Y, Cohen C, Fluss R, Indenbaum V, Amit S, et al. Waning Immune Humoral Response to BNT162b2 Covid-19 Vaccine over 6 Months. N Engl J Med. 2021;385(24):e84.

5. The Updates of COVID-19 in Republic of Korea (March 22, 2023) [press release]. Centers for Disease Control and Prevention Korea, 22 March 2023 2023.

6. Prem K, Zandvoort Kv, Klepac P, Eggo RM, Davies NG, Centre for the Mathematical Modelling of Infectious Diseases C-WG, et al. Projecting contact matrices in 177 geographical regions: An update and comparison with empirical data for the COVID-19 era. PLOS Computational Biology. 2021;17(7):e1009098.

7. Xin H, Wang Z, Feng S, Sun Z, Yu L, Cowling BJ, et al. Transmission dynamics of SARS-CoV-2 Omicron variant infections in Hangzhou, Zhejiang, China, January-February 2022. International Journal of Infectious Diseases. 2023;126:132-5.

8. Liu Y, Zhao S, Ryu S, Ran J, Fan J, He D. Estimating the incubation period of SARS-CoV-2 Omicron BA.1 variant in comparison with that during the Delta variant dominance in South Korea. One Health. 2022;15:100425.

9. Backer JA, Eggink D, Andeweg SP, Veldhuijzen IK, van Maarseveen N, Vermaas K, et al. Shorter serial intervals in SARS-CoV-2 cases with Omicron BA.1 variant compared with Delta variant, the Netherlands, 13 to 26 December 2021. Euro Surveill. 2022;27(6).

10. Zhou F, Yu T, Du R, Fan G, Liu Y, Liu Z, et al. Clinical course and risk factors for mortality of adult inpatients with COVID-19 in Wuhan, China: a retrospective cohort study. Lancet. 2020;395(10229):1054-62.

11. Kim M-C, Cui C, Shin K-R, Bae J-Y, Kweon O-J, Lee M-K, et al. Duration of Culturable SARS-CoV-2 in Hospitalized Patients with Covid-19. New England Journal of Medicine. 2021;384(7):671-3.

12. Lee S, Kim T, Lee E, Lee C, Kim H, Rhee H, et al. Clinical course and molecular viral shedding among asymptomatic and symptomatic patients with SARS-CoV-2 infection in a community treatment center in the Republic of Korea. JAMA internal medicine. 2020;180(11):1447-52.

13. He D, Zhao S, Lin Q, Zhuang Z, Cao P, Wang MH, et al. The relative transmissibility of asymptomatic cases among close contacts. International Journal of Infectious Diseases. 2020.

14. Yin G, Jin H. Comparison of transmissibility of coronavirus between symptomatic and asymptomatic patients: Reanalysis of the Ningbo Covid-19 data. JMIR Public Health and Surveillance. 2020;6(2):e19464.

15. Slifka MK, Gao L. Is presymptomatic spread a major contributor to COVID-19 transmission? Nature Medicine. 2020:1-3.

16. News Y. Average medical expenses of KRW 16 million per critically ill patient with COVID-19 last year (in Korean) 2023. Available from: <https://www.yna.co.kr/view/AKR20230227064900530?input=1195m>. [Accessed April 21, 2023]

17. Jo S, Nam HK, Kang H, Cho SI. Associations of symptom combinations with in-hospital mortality of coronavirus disease-2019 patients using South Korean National data. PLoS One. 2022;17(8):e0273654.

18. Lin DY, Xu Y, Gu Y, Zeng D, Sunny SK, Moore Z. Durability of Bivalent Boosters against Omicron Subvariants. N Engl J Med. 2023;388(19):1818-20.

19. Jo Y, Kim SB, Radnaabaatar M, Huh K, Yoo J-H, Peck KR, et al. Model-based cost-effectiveness analysis of oral antivirals against SARS-CoV-2 in Korea. Epidemiology and Health. 2022;44.

20. Nishiura H, Kobayashi T, Miyama T, Suzuki A, Jung S-m, Hayashi K, et al. Estimation of the asymptomatic ratio of novel coronavirus infections (COVID-19). International Journal of Infectious Diseases. 2020;94:154-5.

21. Kimball A, Hatfield KM, Arons M, James A, Taylor J, Spicer K, et al. Asymptomatic and presymptomatic SARS-CoV-2 infections in residents of a long-term care skilled nursing facility—King County, Washington, March 2020. Morbidity and Mortality Weekly Report. 2020;69(13):377.

22. Kim JE, Choi H, Choi Y, Lee CH. The economic impact of COVID-19 interventions: A mathematical modeling approach. Front Public Health. 2022;10:993745.

23. Nyberg T, Ferguson NM, Nash SG, Webster HH, Flaxman S, Andrews N, et al. Comparative analysis of the risks of hospitalisation and death associated with SARS-CoV-2 omicron (B.1.1.529) and delta (B.1.617.2) variants in England: a cohort study. Lancet. 2022;399(10332):1303-12.

24. Jennifer Kates CC, Josh Michaud. How Much Could COVID-19 Vaccines Cost the U.S. After Commercialization? KFF2023. Available from: <https://www.kff.org/coronavirus-covid-19/issue-brief/how-much-could-covid-19-vaccines-cost-the-u-s-after-commercialization/>. [Accessed June 04, 2023]

25. KDCA. Announcement of vaccination cost according to Regulation on Consignment of Vaccination Service KDCA: KDCA; 2023. Available from: [https://www.kdca.go.kr/board/board.es?mid=a20501000000&bid=0017#](https://www.kdca.go.kr/board/board.es?mid=a20501000000&bid=0017). [Accessed April 04, 2023]

26. Revised Livelihood and Medical Care Support Programs to Be Implemented on July 11 [press release]. Mnistry of Health and Welfare: Mnistry of Health and Welfare, June 29, 2022 2022.

27. Service HIRA. Health insurance self-burden standard guide (in Korean) Health Insurance Review & Assessment Service: Health Insurance Review & Assessment Service; 2023. Available from: <https://www.hira.or.kr/dummy.do?pgmid=HIRAA030056020110>. [Accessed April 21, 2023]

28. Choi E. 1.13 trillion won in treatment costs for COVID-19. 75.1% of health insurance financial statements. 2021 August 02, 2021.

29. Shin G, Kang D, Cheong HJ, Choi SE. Cost-Effectiveness of Extending the National Influenza Vaccination Program in South Korea: Does Vaccination of Older Adults Provide Health Benefits to the Entire Population? Vaccines (Basel). 2022;10(6).

30. Salomon JA, Haagsma JA, Davis A, de Noordhout CM, Polinder S, Havelaar AH, et al. Disability weights for the Global Burden of Disease 2013 study. Lancet Glob Health. 2015;3(11):e712-23.

31. Haagsma JA, Maertens de Noordhout C, Polinder S, Vos T, Havelaar AH, Cassini A, et al. Assessing disability weights based on the responses of 30,660 people from four European countries. Popul Health Metr. 2015;13:10.

32. Bae EY, Hong J, Bae S, Hahn S, An H, Hwang EJ, et al. Korean Guidelines for Pharmacoeconomic Evaluations: Updates in the Third Version. Appl Health Econ Health Policy. 2022;20(4):467-77.

33. Korea) BBo. Arbitraged Rates of Major Currencies Against Won, Longer Frequency Economic Statistics System: Bank of Korea; 2023. Available from: <https://ecos.bok.or.kr/#/SearchStat>. [Accessed July 10, 2023]

34. Consumer Price Index in June 2023: Statistics Korea; 2023. Available from: <https://kostat.go.kr/board.es?mid=a20109020000&bid=11751&act=view&list_no=426208>. [Accessed July 04, 2023]

35. KDCA. COVID-19 Vaccination KDCA: KDCA; 2023. Available from: <http://www.xn--19-9n4ip0xd1egzrilds0a816b.kr/>. [Accessed April 20, 2023]
